# Cytostatics in Indoor Environment: An Update of Analytical Methods

**DOI:** 10.3390/ph14060574

**Published:** 2021-06-15

**Authors:** M. Francisca Portilha-Cunha, A. Alves, Mónica S. F. Santos

**Affiliations:** LEPABE—Laboratory for Process Engineering, Environment, Biotechnology and Energy, Faculty of Engineering, University of Porto, R. Dr. Roberto Frias, s/n, 4200-465 Porto, Portugal; mfcunha@fe.up.pt (M.F.P.-C.); aalves@fe.up.pt (A.A.)

**Keywords:** antineoplastic drugs, cytotoxic drugs, wipe sampling, environmental contamination, surface contamination, air contamination, occupational exposure, healthcare workers

## Abstract

Periodic and adequate environmental monitoring programs are crucial to assess and reduce the occupational exposure of healthcare workers to cytostatics. The analytical methods employed should be rapid, reliable, sensitive, standardized, and include multiple compounds. A critical overview of recent overall procedures for surface and air contamination with cytostatics in workplace settings is presented, with a focus on sampling, sample preparation, and instrumental considerations. Limitations are also addressed and some recommendations and advice are provided. Since dermal absorption is the main exposure route, surface contamination is the preferred indicator of biological uptake and its methods have significantly improved. In contrast, cytostatics’ inhalation is rare; thus, air contamination has been poorly studied, with little improvement. Still, some elements of the analytical methods have not been extensively explored, namely: the amount of wetting solution, the extraction procedure, surface chemistry and roughness, recovery studies from specific surfaces, and cytostatics stability (in surfaces and during shipping and storage). Furthermore, complete validation data (including precision, accuracy, and instrumental and method detection limits) and estimation of global uncertainty are still lacking in most studies, thus preventing method comparison and proposal of standardized procedures.

## 1. Introduction

Cytostatics, also referred as cytotoxic and antineoplastic drugs, have been largely employed in cancer treatment for the past decades, improving cancer survival. However, they are relatively nonspecific, affecting simultaneously malignant and normal cells, which may lead to adverse health effects. In fact, these drugs are potentially carcinogenic, mutagenic, and/or teratogenic to humans [1]. Some are classified by the International Agency for Research on Cancer (IARC) as carcinogenic to humans (Group 1) or probably or possibly carcinogenic to humans (Group 2), while many remain unclassified due to lack of toxicological information [2]. Besides treated patients, healthcare workers, such as nurses and pharmacy professionals, are particularly exposed. Still, other workers connected to the cytostatic circuit (manufacturing, transport, storage, preparation, administration, waste disposal, sanitation), normally less protected, may also be exposed.

Research on workplace contamination with cytostatics has been constantly addressed, and a significant increase in the production of scientific documents over the years has been observed. Numerous guidelines on safe handling of cytostatics have been published [3] and personal protective equipment, ventilated engineering controls, and isolators are generally employed nowadays [4]. Yet, studies worldwide still describe cytostatic contamination in healthcare settings and evidence of adverse health effects related to human absorption (presence in urine and blood samples) [5,6]. Furthermore, no exposure limit values have been set for these drugs since no level of exposure was considered safe. As such, prevention is still based on compliance with guidelines and the “as low as reasonably achievable” (ALARA) principle remains the best standard to reduce occupational exposure.

Periodic environmental monitoring programs are therefore essential and have been largely recommended. Although simultaneous assessment of biological samples would be desirable (informing on actual human uptake), it can be a lengthier practice due to the need for ethical approval and to conform to specific requirements and guidelines. Besides, environmental monitoring already allows identification of the exposure risk, implementation of preventive measures and validation of decontamination practices. Since dermal absorption by direct skin contact with contaminated surfaces and equipment is the primary way of exposure [7], surface contamination is the best predictor of possible biological contamination and has been investigated more frequently. In contrast, air contamination raises fewer concerns due to the widespread use of Biological Safety Cabinets (BSCs) and proper protective measures. In fact, recent studies on that topic are scarce and most follow up on work developed from the 1980s to the early 2000s. However, air contamination in the workplace might occur due to improper handling of BSCs or to accidental events, mainly in locations where cytostatics are manipulated under pressure (which may result in the release of aerosols) [7,8]. Hence, analysis of airborne particulate matter and aerosols might be relevant sometimes.

Environmental monitoring may be divided in five main phases: planning a monitoring strategy; sampling (and storage); sample preparation; sample analysis; and interpretation (and reporting) of results. In fact, the whole process is dependent on the reasons for conducting it, as well as its objectives—which should be considered beforehand [9]. For example, the locations sampled and the number of samples for hazard identification will likely differ from those for evaluation of engineering and administrative controls. To ensure an adequate monitoring program, rapid, reliable, and standardized sampling techniques and analytical methods are required. Nonetheless, no unanimous method has been developed, owing to various reasons: the use of very distinctive compounds in chemotherapy; the demand for increasing method sensitivity; or the usual economic constraints, to name a few. In fact, new articles trying to overcome such obstacles, thus optimizing these methods, keep surfacing. Furthermore, lack of validation makes it challenging to compare results obtained from different methods, thus limiting accurate quantification and exposure risk assessment.

Previous reviews on this topic exist, but they comprise data published up to 2013 and have mostly focused on surface contamination and on wipe sampling methodologies [5,7,9]. Besides, they generally compare overall sampling and extraction procedures of different studies, rather than discussing the relevance and impact of each technical factor or variable on the overall analytical method performance. Therefore, we aim to review recent analytical methods specifically developed for sampling, sample preparation, and instrumental assessment of cytostatic contamination of surfaces and air from workplace settings. We further intend to discuss present limitations, provide some guidance, and propose recommendations. Attention will also be given to validation procedures and overall method considerations. Planning a monitoring strategy will not be deeply discussed, except for cytostatics’ selection within the studies under review; neither will the interpretation of results.

## 2. Literature Search

A thorough literature search was performed on the common literature database SCOPUS, with multiple combinations as search terms. “Antineoplastic”, “cytostatic”, “anticancer”, “antiblastic”, and “cytotoxic” were each combined with relevant keywords, namely: “environmental monitoring”, “wipe/wiping”, “occupational”, “work surface”, “surface contamination”, “air” and “on-site/onsite”. A total of 35 combinations were employed. Additionally, only original articles written in English and published after 2013 were retained. That year was chosen because previous reviews have already scrutinized former original articles on the topic [5,7].

Duplicates were removed and a first selection was based on the title. Further screening was performed after abstract analysis and 24 articles remained. However, after contents were checked to validate their relevance, four publications were discarded—their focus was on developing analytical methods for better production of pharmaceutical formulations rather than assessing contamination in work environment.

From the aforementioned search, 18 articles were categorized as analytical methods: 16 for surface contamination [10,11,12,13,14,15,16,17,18,19,20,21,22,23,24,25] and two for air contamination [26,27]. The other two articles will not be further examined here as they refer to non-analytical methods for surface contamination assessment [28,29].

## 3. Cytostatics Included in the Studies

Previously, most studies would focus on a few surrogate cytostatics, i.e., indirect indicators for the presence and quantity of all cytostatics expected in a surface. Those were selected based on: their wide application as antineoplastic drugs; their frequent use in current chemotherapy preparations, as stated by the hospital unit involved in the study; the existence of previous studies in the literature regarding similar research projects developed in other countries; their classification by IARC [2]; among other reasons. Nowadays, although the same rationale is applied for their selection, the number of cytostatics included in the methods being developed has increased. This is both a consequence of the emergence of more selective methods (which are able to simultaneously identify several compounds) and of the increasing notion that surrogate cytostatics do not provide a comprehensive picture of the exposure risk (because individual physicochemical characteristics impact cytostatics’ sorption on surfaces or air dispersion and because each one has considerably different impacts on health).

In fact, of the 16 articles describing analytical methods for surface contamination, only four examined less than five cytostatics, while five studied more than 20. As reported in Table 1, a total of 31 cytostatics were evaluated in those articles, namely: cyclophosphamide (CYC, studied in 15 articles); 5-fluorouracil (5FU, 12); doxorubicin (DOX, 12); ifosfamide (IFO, 12); paclitaxel (PAC, 11); docetaxel (DOC, 10); methotrexate (MET, 10); epirubicin (EPI, 9); gemcitabine (GEM, 9); irinotecan (IRI, 8); vincristine (VCR, 8); cytarabine (CYT, 7); etoposide (ETO, 7); dacarbazine (DAC, 6); topotecan (TOP, 6); vinblastine (VBL, 6); idarubicin (IDA, 5); mitomycin C (MIT, 4); pemetrexed (PEM, 4); busulfan (BUS, 3); daunorubicin (DAU, 3); etoposide phosphate (ETP, 3); fludarabine phosphate (FLU, 3); raltitrexed (RAL, 3); fotemustine (FOT, 2); ganciclovir (GAN, 2); melphalan (MEL, 2); oxaliplatin (OPt, 2); vindesine (VDE, 2); vinorelbine (VOR, 2); and carboplatin (CPt, 1); platin (Pt) was also used as a marker of cis-, carbo- and oxaliplatin in two studies. The prevailing reasons for inclusion were their common/frequent use in chemotherapy preparations [11,12,14,20,22,23] and a requirement to include several pharmacological classes in the methodology being developed [11,15,16,20,21,25]. Some also based their choice on IARC classification [10,19,25], on partner hospital’s practice [10,13,25] and whether there existed previous studies or documented toxicity regarding proposed cytostatics [10,11]. Still, three articles did not provide any rationale for their choices [17,18,24]. Table 1 also presents the chemical structures of the mentioned analytes.

Regarding the two articles that developed methods for air contamination assessment, only CYC was analyzed in both cases. In fact, it seems that the most reliable data in the literature from air contamination pertains to CYC [30]. The lack of studies in this topic along with the reduced number of cytostatics they evaluated may be explained by the fact that most cytostatics are not reportedly volatile. However, according to these articles, vaporization phenomena leading to both gaseous and particle forms of CYC have been previously reported. Moreover, it was frequently used at partner hospitals.

## 4. Surface Contamination

**Figure 1:**
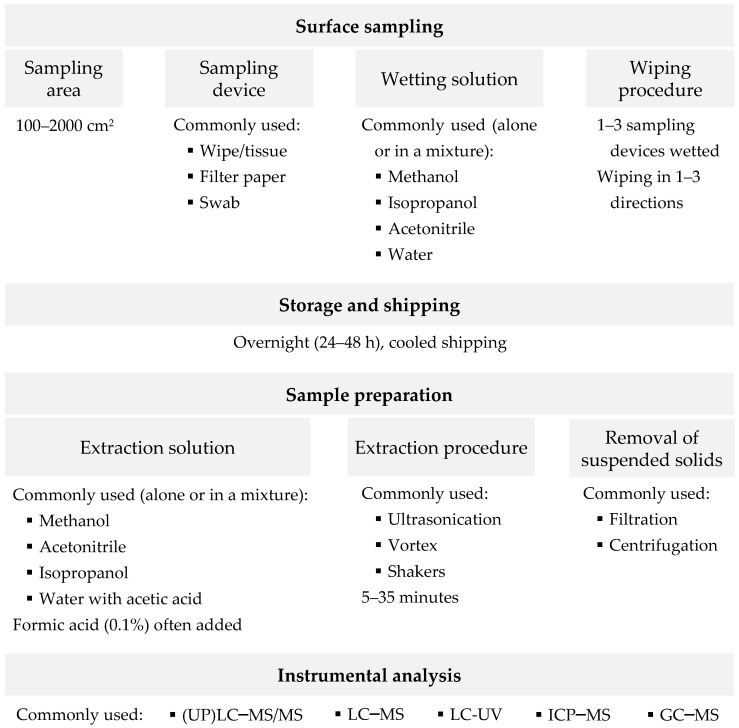
Schematic representation of the main considerations for the development of an analytical methodology to monitor the contamination of surfaces by cytostatics. (UP)LC–MS/MS—(ultra-performance) liquid chromatography with tandem mass spectrometry; LC–MS—liquid chromatography with mass spectrometry; LC–UV—liquid chromatography with ultraviolet detector; ICP–MS—inductively coupled plasma mass spectrometry; GC–MS—gas chromatography with mass spectrometry.

A schematic representation of the main considerations for the development of an analytical methodology to monitor the contamination of surfaces by cytostatics, which are discussed in the next subsections, is shown in Figure 1.

### 4.1. Sampling

Regarding sampling, Table 2 displays the main sampling elements employed for the analytical methods for surface contamination under review. The most relevant are the type of sampling devices and the wetting solution since both largely impact cytostatics’ extraction efficiency from surface material. Moreover, sample preservation and stability during storage will also be addressed, as these considerations are highly dependent on the sampling procedure and should always be pursued and ensured.

#### 4.1.1. Surface Type and Area

Cytostatics’ recovery has been shown to be dependent on the specific type of surface sampled [9]. Moreover, method application in monitoring programs of healthcare settings typically require sampling of diverse surfaces. Hence, recovery studies on surfaces made of different materials are crucial to correctly provide and interpret results. However, as observed on Table 2, many studies only develop their methods for one surface, specifically stainless steel, while many do not even report the surfaces used. Although some articles indicate that recovery studies have been carried out on surfaces made of different materials, most of them do not provide the recoveries from the surface but from the sampling device (e.g., swab, filter paper) [16,21,24,25]. On the other hand, the few studies that provide recoveries from surfaces present a single recovery for each cytostatic, independent on the surface type [11], except for the study of B’Hymer and co-workers [22]. They studied the wipe recovery of MIT from three different surfaces and found that stainless steel and Formica^®^ generally had higher recovery yields (ranging from 61–98% and 63–97%, respectively) than vinyl surfaces. Vinyl is a porous material, whose recovery yields were of 50–60% [22].

The first study addressing the effect of the state/condition of the surface on cytostatics recoveries was performed by Jeronimo and co-workers in 2015. The authors verified a notable increase in the recoveries of six cytostatics from new stainless steel plates versus worn plates (45% to 80% vs. 10% to 59%) [23]. The lower recoveries on older plates were attributed to the physically worn surface, which indeed led to higher surface roughness (95.8 ± 10.9 and 290.8 ± 50.7 for new and old plates, respectively), making effective wiping more difficult [23]. An extensive study to examine the variation of cytostatics recoveries based on the surface roughness was carried out by Colombo et al. [17]. It was confirmed that there is a decrease on cytostatics recoveries with an increase in the stainless steel plate roughness and that roughness plays an important role in determining the recovery of wipe sampling [17]. However, other parameters (e.g., surface chemistry) were found to also be important to the overall process performance, as a large variation in cytostatics recoveries (most noticeably with 5FU and OPt) was observed within plates with similar surface roughness [17]. Actually, cycles of cleaning using various solvents and/or heating may affect surface chemistry, without affecting surface roughness.

These results highlight the importance of gathering ancillary information (e.g., surface roughness, throughput, timing, cleaning procedures) to help interpret the analytical results. In fact, false-negatives or underestimation of cytostatics’ concentration on rough, more used or porous surfaces may hinder a full assessment of drug contamination and exposure in healthcare facilities. Hence, it may constitute an issue of concern in terms of public health and safety. In order to overcome these problems, the analyst may decide to perform a second wipe on the same sampled area, but this must be carefully planned as some disadvantages are foreseen (more details can be found in Section 4.1.4.).

Considering the sampling area, most studies under review sampled 100 cm^2^ of target surfaces (and never less than that). However, as surface contamination has typically been reported in the level of ng/cm^2^ or even pg/cm^2^, sampling a larger area might be beneficial. This would likely increase the amount of cytostatics recovered per sample, thus avoiding that samples fall near the instrumental detection limits (which would prevent their detection and quantification). In fact, some studies already sampled much higher surface areas: up to 2000 cm^2^ (Table 2). It is also worth noting that most studies indicate a preference for regular, planar surfaces, since areas of uneven surfaces are not so easily determined. In such cases, results are typically provided as an absolute amount (mass) per sampling device or per object (e.g., glove, doorknob), rather than the area being estimated.

#### 4.1.2. Sampling Devices

As can be seen in Table 2, three main sampling devices have been used to extract cytostatics from surfaces: wipes/tissues [11,16,18,20,23], cellulose filter papers [10,14,17,19,22,23,25], and swabs [14,21,22,24].

Regarding the wipes/tissues, the brands are indicated (KIMTECH Science Precision [18,20,23], Kleenex [11], and STS Medical Group Luigi Salvadori [16]), but the product details (e.g., composition, grammage) are not specified. Moreover, recoveries of cytostatics from surfaces are barely reported, making the comparison between wipes/tissues impossible in terms of removal efficacy. However, Jeronimo and co-workers referred that KIMTECH Science Wipers (#05511) interfered with the detection of 5FU, and that this interference was detected in brand new, unspiked Kimwipes [23]. Since 5FU is one of the most prescribed and handled cytostatics, the use of wipes/tissues as sampling devices has a relevant limitation.

The cellulose filter papers (Whatman 42, 55 mm) are by far the most used sampling devices. However, Guichard et al. compared the wiping efficiency of 23 cytostatics from stainless steel using cellulose filter papers and swabs made of different materials [14], and found that cellulose filter papers exhibited the lowest recoveries for most of the cytostatics (mean recoveries of 69% with either sonication or vortexing desorption modes). Moreover, the authors reported that the handling of filters is not ideal, as they require the use of tweezers to hold them and they have low resistance to abrasion (easily disintegrate when surfaces are not smooth) [14]. The swab made of polyurethane yielded 74% and 84% recoveries for sonication and vortexing, respectively, and was only advantageous for anthracycline cytostatics (DOX, EPI, IDA, DAU), and ETP in both desorption modes. Polyester swabs gave globally higher recoveries for all cytostatics with mean values of 90% (TX714) and 95% (TX716), regardless of using sonication or vortexing. Besides the slightly higher average recovery with TX716, this polyester swab is indicated as clean room-compatible by the manufacturer [14].

B’Hymer and co-workers compared the extraction efficiency of MIT from different surfaces (stainless steel type 304, vinyl and Formica^®^) using a cellulose filter paper and a polyester swab [22]. There were little differences in using filters or polyester swabs for extracting MIT from stainless steel or vinyl surfaces. The main quantitative advantage of using the polyester swab over filter paper was found for the Formica^®^ surface at the lowest spiking levels. The polyester swabs had recovery yields of 60% to 70% at the 0.1 and 0.2 µg/cm^2^ levels while the filter paper had near 30% [22]. Nonetheless, qualitative advantages were reported for swabs: they would not crumble or tear during the wiping procedure and tended to cause less physical hand fatigue [22].

Acramel et al. also described that wiping and desorption rates were better when employing viscose swabs in comparison with Kimwipe Science Precision Wipes and Kimwipe Science Delicate Wipes (especially for PEM, DOC, and MET) [24]. Furthermore, this study noted that swabs reduce the risk of contamination since only a small fraction (the tip) contacts with the surface.

Therefore, swabs are generally good wipe sampling devices and, so far, they seem the most suitable for the intended purpose. On the other hand, filter papers are not recommended, despite its frequent use in the past.

#### 4.1.3. Wetting Solution

The nature, the composition and the amount of wetting solution has a strong effect on the global recovery of cytostatics. The wetting solution must be suitable for sampling both polar and nonpolar compounds from surfaces (cytostatics have a wide range of log D, i.e., the water:octanol partition coefficient at a specific pH), ensuring a sufficient transfer of cytostatics from surface into the sampling device [18]. Furthermore, this solution is directly in contact with the sampled surface made of different materials and must not alter them. Moreover, a non-toxic solution is mandatory since the wiping procedure is performed in hospitals by its own staff. Therefore, the choice of solvent is mainly restrained to those already used for microbial decontamination, for instance the alcohol-based solvents isopropanol and methanol (MeOH). Still, water (H_2_O) and acetonitrile (ACN) have also been employed, as observed in Table 2.

Jeronimo et al. studied the impact of different wetting solutions on the recovery of 6 cytostatics from stainless steel surfaces: H_2_O, H_2_O/MeOH [80:20, 50:50, and 20:80], MeOH, and H_2_O/ACN/MeOH [65:10:25], all acidified with 0.1% formic acid [23]. They concluded that CYC, Opt, and 5FU were not significantly affected by the chemical composition of the wetting solution (standard deviations lower than 8%). However, the recoveries of MET were found to increase with the aqueous percentage on the wetting solution: maximum recovery of 40% for H_2_O/ACN/MeOH [65:10:25] and minimum recovery of 6% with MeOH [23]. An opposite trend was observed for VCR and PAC, whose recoveries increased with the organic solvent percentage on the wetting solution. The highest recoveries were 65% for VCR using MeOH and 85% for PAC using H_2_O/MeOH [20:80]. Guichard and co-workers also compared the wiping efficiency of wetting solutions composed of different concentrations of isopropanol on the recovery of 23 cytostatics from stainless steel surfaces. The best recoveries for all cytostatics were obtained with 75% isopropanol, an intermediate situation was achieved with 25% isopropanol, while the lowest recoveries were found with 100% isopropanol [14].

The amount of wetting solution is a poorly addressed parameter, but its investigation is advised and encouraged, as it may strongly affect the efficiency of the wiping step. In fact, only one study addressed the importance of optimizing this parameter: Jeronimo et al. found that moistening cellulose filter papers with 0.8 mL instead of 0.5 mL of solvent led to difficulties in handling the filters, compromising the extraction process [23]. Table 2 shows that volumes from 0.05 to 10 mL were used, though no justification to choose such values are given in any study. Nonetheless, the choice of wetting solution volume should take into consideration the sampling area and the type of sampling device used.

#### 4.1.4. Wiping Procedure

The wiping procedure is another relevant parameter affecting the overall recovery of cytostatics from surfaces. Table 2 evidences that most studies use between one and three wet sampling devices for this procedure (since a dry sampling medium would have little tendency to extract cytostatics from surfaces). A few studies further add a final step with a dry sampling device to pull the solvent that may have remained on the surface. Yet, B’Hymer et al. used only dry filters after applying the wetting solution directly on the surface [22]. No attempts have been made to compare recoveries from each technique, but the latter cannot be applied to irregular surfaces, severely limiting its implementation. Although most studies do not give specific indications for their wiping procedure, some indicate that wiping should be performed in two or three directions: usually horizontal, vertical and diagonal or right, left and down. According to Hetzel and co-workers, one sampling device should be used for each direction and it should be wiped in a perpendicular direction once (at the end of each step) to avoid accumulation of the analytes on one side of the sampling area [18].

Different procedures involving wiping the surface twice were tested by Jeronimo and co-workers [23]. The second wiping, using a cellulose filter moistened with H_2_O/MeOH [80:20 or 50:50] with 0.1% formic acid, did not significantly increase the recoveries of most drugs, except MET and CYC. For these, a maximum of 16% and 19% increase was recorded with H_2_O/MeOH [80:20] with 0.1% formic acid, respectively [23]. On the other hand, wiping twice with the same filter yielded recoveries inferior to wiping once (9, 21, 15, 20, 22, and 23% less for 5FU, OPt, MET, CYC, VCR, and PAC, respectively). The authors attributed these results to the deterioration of the filter, as residues of the filter were observed on plate surface after wiping the second time [23]. Wiping twice, each time applying 125 μL of H_2_O/ACN/MeOH [65:10:25] with 0.1% formic acid directly on the stainless steel plate and using a dry filter, only increased the recovery of MET in 18% [23]. The same conclusion was reported by Colombo and his team [17], i.e., wiping again with the same filter yielded lower recoveries likely due to physical degradation of the sampling device, especially on a rough surface, and a second (new) wipe only yielded an additional 10% average recovery.

Considering these findings and that a second wipe step doubles the time and cost of analysis, it seems reasonable to advise on the use of one wiping step only. Furthermore, bearing in mind that the wiping procedure is meant to be applied in healthcare facilities, where numerous surfaces are irregular/non-horizontal (e.g., doorknobs, bottles of drugs), the wetting solution should be applied on the sampling devices rather than directly on surfaces.

#### 4.1.5. Wiping Personnel

The wipe sampling procedure aiming at collecting cytostatics from surfaces in hospitals or healthcare centers are likely to be carried out by different personnel working at the facilities. Therefore, it is crucial to know the extent to which the recovery variability depends on the personnel who take the sample. Jeronimo et al. evaluated the wiping recoveries among different personnel and found a considerable variability for all cytostatics studied [23]. Furthermore, they verified that the highest recoveries were consistently obtained by experienced personnel. Contrarily, the method proposed by Colombo and co-workers yielded good recoveries in real-case scenarios (multiple personnel collecting over multiple days) for every target cytostatic [17]. In any case, these findings suggest that external personnel training is recommended, by providing instructions and detailed demonstration of the wiping method, to ensure that more accurate results are achieved.

#### 4.1.6. Stability of Cytostatics on Surfaces

Previous surface wipe assessments have suggested that CYC and IFO may be stable on surfaces [33,34], but this phenomenon was only explored in depth, with these and other cytostatics, by Colombo et al. in 2017 [17]. The research team evaluated the long-term behavior of 10 cytostatics on the stainless steel over a period of 1 month, without exploiting the process that may affect the recovery of the drugs over time (e.g., photodegradation, adsorption, surface chemistry). OPt, VDE, VCR, and VBL exhibited a sharp decrease in their recoveries after 24 h (by ~20% versus ~85% initially), remaining constant close to the instrumental detection limit (IDL) for 1 month. Another pattern was observed for 5FU, CYC, MET, and IFO, whose recoveries decreased steadily, declining to values of ~20% after two weeks. DOC and PAC were revealed as the most stable: the recovery of DOC smoothly decreased, reaching values of 40% after three weeks, after which it stayed nearly stable; and the recovery of PAC began to decrease in a slow fashion after a week, being ~80% after a month.

These results highlight the importance of knowing the last time that each drug was manipulated in the setting under evaluation to accurately evaluate the contamination of the indoor environment by cytostatics through surface wiping. This is particularly relevant for the most stable cytostatics on surfaces, such as DOC and PAC. These could experience a cumulative effect on the surfaces, through successive contaminations, reaching higher concentrations, and increasing the probability of a surface being contaminated at dangerous levels. Actually, persistent cytostatics are very likely to be spread to different departments/areas in healthcare facilities (from reception to disposal).

#### 4.1.7. Storage and Shipping

The shipping of the samples from the hospitals to the laboratories and the storage conditions are two key steps. However, few studies have been performed to understand the behavior of cytostatics during shipping/storage processes. The stability of six cytostatics (CYC, 5FU, MET, OPt, PAC, and VCR) on wiping filters was firstly evaluated over six days at 5 °C, by Jeronimo et al. in 2015 [23]. During the first 48 h, the degradation of cytostatics was relatively low, except for VCR (9% degradation). No significant differences on recoveries of CYC, 5FU, MET, Opt, and PAC were also observed in the real-case shipping assay (48 h at a mean temperature of 13.6 °C) [23]. However, an increase in the degradation was observed after 6 days at 5 °C, with significant differences for all drugs except MET and CYC [23]. The stability tests performed by Colombo and co-workers also revealed that no significant degradation of 10 cytostatics on wiping filters occurred during the first 24–48 h at 5 °C [17]. Moreover, no significant change was observed in 8 out of 10 cytostatics after one week; 5FU and IFO had higher recoveries after being stored for a week [17]. A similar behavior for 5FU was also reported by Tuerk et al. [35]. Müller-Ramírez and his team investigated the stability of CYC, IFO and PAC on wiping filters at −20 °C and on solid-phase extraction (SPE) cartridges at 20 °C for a period of two months, concluding that no significant decrease of recoveries were observed [10].

Considering recent data on the stability of cytostatics, the best shipping option is an overnight (24–48 h) cooled shipping. Furthermore, it seems that SPE cartridges might provide a safer way to handle wipe samples containing cytostatics, in the event that samples cannot be analyzed immediately, or when they need to be shipped to other laboratories for more complete analysis.

### 4.2. Sample Preparation

#### 4.2.1. Extraction Solution

Table 2 also displays the main parameters to take into consideration for sample preparation. In this phase, the extraction solution and procedure are the most relevant.

The nature of the solvent used to desorb compounds from the sampling device to the solution is a key factor to obtain satisfactory recoveries. The OSHA evaluation guidelines for surface sampling recommend a minimum recovery of the analyte from the sampling device of 75% [36]. Moreover, the solvent must be compatible with the initial conditions of the gradient used in the liquid chromatography tandem mass spectrometry (LC–MS/MS) method. The most common solvents are MeOH, ACN, isopropanol, and water acidified with acetic acid. Some solutions also include dimethyl sulfoxide (DMSO), potassium phosphate buffer, and ethyl acetate, and a few add 0.1% formic acid to the extraction solution.

To the authors’ best knowledge, only two of the target studies of this review investigated the effect of different solvents in the extraction of cytostatics from the sampling devices. One of the studies indicated that no significant differences were observed between using H_2_O/MeOH [80:20 and 50:50], both acidified with 0.1% formic acid, to extract CYC, 5FU, MET, OPt, PAC and VCR from cellulose filter papers [23]. However, the authors decided for the extraction solvent composed of H_2_O/MeOH [50:50] due to LC–MS/MS compatibility with high injection volumes [23]. The study from Hetzel and co-workers investigated the effect of different compositions of DMSO, isopropanol or ACN with water for the extraction of 11 cytostatics from wipes [18]. The results demonstrated that all cytostatics could be extracted sufficiently with a portion of 20% DMSO in water, except DOX and EPI. The increasing content of ACN (5% to 20% in 5% steps) in water led to increasing recoveries of DOX and EPI, anticipating that an even higher ACN concentration could lead to recoveries higher than 75%. However, this option was not considered since serious chromatographic problems for polar compounds would be found, due to the missing focusing on the column head (e.g., GEM was not recovered for ACN contents higher than 10%) [18]. Finally, isopropanol/water [30:70] was identified as the best extraction solvent, with recoveries ranging from 80% to 120% for all cytostatics. The protic character of isopropanol favors the extraction of DOX and EPI due to the possibility of hydrogen bonding and solvation [18].

The results of Rossignol et al. further show how difficult it is to consider several aspects when choosing the extraction solution: in trying to limit inhalation exposure of lab technicians, by excluding the use of organic solvents, they ended up obtaining very low recoveries for four of the six cytostatics studied [25], thus compromising the efficiency of their method.

#### 4.2.2. Extraction Procedure

Table 2 shows that all studies employ an agitation step during sample preparation, which last between 5 and 35 min. Ultrasonication is the most common, but vortex and shakers have also been used, as well as an automated wipe desorption device.

Like the previous parameter, the extraction procedure has been poorly studied. Only Guichard et al. compared two different techniques: sonication and vortex (for the same extraction time) [14]. However, these results were not discussed in depth since no significant differences on mean recoveries were found for the two polyester swabs (95% for TX716 and 90% TX714) and the cellulose filter paper (69%), and because the difference for the polyurethane swab (TX712) was low (84% for vortex, 74% for sonication). Indeed, the only observation on individual recoveries was that DOC and PAC were less than 10% recovered with the polyurethane swab using sonication. Regarding extraction time, Jeronimo et al. compared 20 and 35 min of sonication, and concluded that no significant differences were observed for any of the six cytostatics studied [23]. Such lack of studies focusing on extraction conditions further highlights that methods for surface contamination with cytostatics might still be improved.

Additionally, while for some studies the sample preparation phase consists only of the extraction technique, others employ a final step to remove suspended solids prior to instrumental analysis. Filtration and centrifugation are both equally used. Furthermore, one study employed SPE, evaporated the sample (under nitrogen) to complete dryness and reconstituted it in an appropriate solvent. However, such steps might have a negative impact on recovery. For example, B’Hymer et al. decided to employ a filtration step (with a polyvinylidene fluoride filter) for sample pre-concentration, since using solvent evaporation significantly degraded MIT; Colombo et al. disclosed that a final syringe filtration step was excluded from their original method to improve the desorption efficiency [17]. Yet, no quantitative analysis was presented in any case. Hence, assessment of every extraction step (namely, the impact on recovery efficiency), which generally has not been performed, should be contemplated.

#### 4.2.3. Stability of Sampling Extracts

Rossignol and co-workers investigated the stability of extracted solutions when kept in the instrumental rack inside the autosampler for 24 h at 15 °C, and they concluded that the estimated loss of six cytostatics (CYC, DOX, EPI, 5FU, GEM, IFO) was lower than 15% [25]. Good stability of CYC, DOX and 5FU was also observed in autosampler stability studies conducted by Bobin-Dubigeon et al. under the same conditions (24 h at 15 °C). However, the studies after 48 h showed instability of the extract solution [19]. Likewise, Acramel et al. showed that, of the 14 cytostatics studied (5FU, CYC, CYT, DAC, DOC, DOX, EPI, ETO, GEM, IFO, IRI, MET, PAC, PEM), all were stable (relative bias below 15%) at 4 °C after 24 h, and that only DAC was not stable at ambient temperature during the same time [24]. MIT was also stable in the autosampler at 8 °C for 8 days (4% degradation) [22].

Regarding the effect of freeze/thaw processes, Bobin-Dubigeon et al. found that CYC, DOX and 5FU were stable after 1 week and after 1 month at −80 °C [19]. Acramel et al. also studied the long-term stability of several cytostatics at −20 °C and concluded that: 5FU, CYT, DOC, ETO, GEM, MET, PAC and PEM were stable up to 60 days (relative bias below 15%); DOX, EPI and IRI were stable up to 30 days (but not 60 days, where deviations were above 23%); and CYC and IFO were not stable for 30 days (relative bias above 23%). However, Rossignol et al. verified that extracted solutions of CYC, DOX, EPI, 5FU, GEM, and IFO were stable for 24 months at −20 °C (coefficient of variations lower than 8%), but these must be analyzed within 24 h after thawing [25]. Such differences in the stability of cytostatics (namely, CYC, DOX, EPI, and IFO) might be related to the use of different solvents.

Nonetheless, the different physicochemical properties of cytostatics (especially when simultaneous detection is intended) could also have a relevant impact. In fact, Dal Bello et al. found good stability at room temperature after 4 h for 5FU, CPt, CYC, CYT, DOX, GEM, IFO and MET, but not for MIT [21]. According to B’Hymer et al., MIT can be easily hydrolyzed under acidic or basic conditions [22]. Hence, using MeOH in the presence of formic acid might have compromised the stability of MIT, although being suitable for the remaining cytostatics. B’Hymer et al. further demonstrated that MIT is light sensitive: it was 12% degraded after 8 days at room temperature when exposed to window sunlight and laboratory fluorescent lights, compared to 3% degraded when kept in darkness [22].

In view of these findings, it is plausible to advert for the need to analyze the extracts within 24 h at 15 °C or 24 h after thawing and for the preferential use of amber containers (to avoid possible light degradation). Furthermore, although cytostatics seem generally stable across these studies, proper stability assessments should always be conducted.

## 5. Air Contamination

### 5.1. Sampling

Cytostatics’ quantification in the occupational air can be achieved through extraction and analysis of suitable sampling devices placed in either passive or active air samplers. Conventional methods to assess pollutants in air are mostly based on active sampling, using paper or glass fiber filters to capture airborne particles. Indeed, most previous methods described for air contamination with cytostatics have followed that procedure [26]. However, CYC has been found to sublimate or evaporate off the filter (as air continues to be pulled through it) [37]; thus, some authors have developed alternative methods using solid sorbents to collect CYC as a vapor [37,38]. More recently, Panahi et al. considered the use of novel methods for sampling and analysis of low concentration of contaminants: solid phase microextraction (SPME) and needle trap devices (NTD). They opted for NTD since it uses active sampling (while SPME requires passive sampling), which may contribute to higher precision, besides being user-friendly and fast [26]. On the other hand, Wakui et al. decided to use a high-performance VOC-SD passive air sampler to overcome problems associated with active samplers, such as difficulty in equipment installation and sampling noise, and because they are easy to carry (which is relevant for personal sampling) [27]. Yet, both studies used carbon molecular sieves as adsorbents.

Other relevant parameters when sampling air are sampler location; total volume sampled; flow rate; and sampling time. Regarding location, both articles mention the importance of putting the sampler near the breathing zone of potentially exposed staff, following indications of previous studies, thus aiming for personal sampling. Indeed, the passive sampler from Wakui et al. was fixed on the surface of the preparer’s mask [27]. Concerning the remaining parameters, although important to obtain concentration values in mass per air volume, allowing comparison between studies, information is sometimes scarce. Despite providing results in those units, Panahi et al. only specify that 300 mL/min was selected as the proper sampling pump flow rate (sampler needle flow rate was measured at 20 mL/min), and do not indicate sampling time nor total volume sampled [26]. It is worth noting that such flow rate is lower than most previously reported in the literature [7,38]. In contrast, Wakui et al. neither specify values for such parameters nor report concentrations in mass per air volume, although mentioning that the sampling time during method application was dependent on the preparation time (which was around 20 min) [27]. Since the amount of drug recovered is dependent on the cumulative volume of air sampled, it seems imperative to further assess the impact of sampling time and volume, and to propose standard values/procedures.

Similar to methods for surface contamination, storage is also poorly addressed in these studies. It is only stated that samples from NTD were kept at −4 °C in a cold box containing dry ice [26], with no mentions to storage time or stability.

### 5.2. Sample Preparation

The same considerations and recommendations mentioned for the preparation of surface samples can be generally applied for samples from air sampling, since the methods used are usually very similar. In fact, most methods found in the literature add an extraction solvent to the sampling device and employ an agitation step prior to instrumental analysis [7]. Besides, some also describe the use of a sample concentration step (SPE or LLE), since sample enrichment might be essential due to the very low values expected for air contamination. Some studies further employ a step to remove suspended solids (filtration or centrifugation) and/or a step of evaporation to dryness followed by reconstitution.

In the study of Wakui et al., that generic approach was followed: the carbon molecular sieve was placed in 1 mL of carbon disulfide (desorption solvent) for 5 min; then removed and placed in 1 mL of water (extraction solvent), which was stirred; LLE was performed; and the solvent was centrifuged [27]. Although three parameters were optimized (desorption solvent, desorption time, LLE solvent), no recovery values are given, except for the indication that near 100% CYC was recovered using the final conditions. In contrast, the study of Panahi et al. did not need to perform sample preparation because the NTD was directly inserted into the injection port of a gas chromatographer [26]. Although desorption time and temperature were optimized (30 s at 300 °C), there is no indication on how performance was evaluated.

Recovery and retention efficiencies are the percentage of analyte recovered from the spiked sampling device and the percentage of analyte retained on the spiked sampling device after a determined air volume was pulled through it, respectively. These values are essential to interpret the results obtained in real applications and to allow comparison between existing methods. Yet, their evaluation has been inadequately explored.

## 6. Instrumental Method of Analysis

Instrumental techniques used for environmental monitoring (surface and air contamination) of cytostatics by the studies under review are shown in Table 3.

The instrumental technique of choice for the determination of cytostatics in surface extracts is LC–MS. Gas chromatography followed by mass spectrometry (GC–MS) was also used, albeit to a much lesser extent, and only for the determination of 5FU, CYC, and IFO [5]. Although comparable IDLs could be obtained with both techniques, LC avoids the derivatization of little volatile compounds (cytostatics generally have high molecular weight) and the drawbacks related with sample pretreatment. Inductively coupled plasma with mass spectrometry (ICP–MS) or voltammetry is sometimes used as a marker of platinum-containing cytostatics [5]. Of the reviews under study, only Dugheri et al. employed this method [15,16], while a few studies have included CPt and OPt in LC–MS/MS methods [17,21,23]. Recently, LC–MS/MS has become an important ally in the determination of many cytostatics at ultra-trace levels in a single run, and most studies already employ this method (Table 3). Actually, the simultaneous analysis of the higher possible number of cytostatics is of paramount importance in this context, due to the high variety of cancer treatments, which often include more than one cytostatic.

For the determination of cytostatics in air, LC–UV has mostly been used, although LC–MS/MS is also common [7]. In fact, Wakui et al. used both methods since the values obtained with LC–UV were close to the IDLs; thus, LC–MS/MS was necessary to allow quantification [27]. In the study of Panahi et al., a less conventional instrumental method called gas chromatography with electron capture detector (GC–ECD) was used as a consequence of the sampling method chosen [26].

### 6.1. Separation of Critical Cytostatics

This section was reserved for the discussion of those critical peak pairs of cytostatics, which require special attention regarding their unequivocal identification and quantification, even when a MS detector is used.

One example is the epimers DOX and EPI, whose identification is only possible based on different retention times (chromatographic separation), because they have identical mass-to-charge ratio (*m*/*z*) transitions and, therefore, cannot be differentiated by MS [18]. CYC and IFO constitute another critical pair. They are isomers and, although different *m*/*z* transitions could be selected for their monitoring, their chromatographic separation is recommended due to cross talk, as demonstrated by Hetzel and co-workers [18]. In addition, the separation of PAC and DOC is deemed necessary because of ion suppression. Moreover, the simultaneous analysis of polar (e.g., 5FU) and non-polar (e.g., PAC) cytostatics is a big challenge and has triggered the attention of the scientific community. Hetzel and his research team demonstrated that the three critical peak pairs (DOX/EPI, CYC/IFO, and PAC/DOC) could be satisfactorily separated in the column YMC Triart C18, using water and ACN, acidified with 0.1% of formic acid, as mobile phase. They also verified that the use of MeOH as organic phase generally led to an increased selectivity, but they decided for ACN because it allowed obtaining sufficient selectivity for the critical pairs, while reducing the time of analysis, thanks to the reduction of viscosity/pressure drop and the increase of the flow rate [18]. Dal Bello et al. also faced the challenge of monitoring CYC and IFO in the same analytical run and concluded that despite the partial coelution of the isomers, their quantification was not affected due to the selection of different product ions (*m*/*z* 140.0028 and *m*/*z* 182.0132, respectively) [21]. Acramel and co-workers developed an analytical methodology for the quantification of 14 cytostatics in surface extracts, with the three critical pairs of cytostatics and 5FU among the target analytes [24]. They found that Acquity UPLC^®^ BEH phenyl column did not allow separation of the isomers within 10 min. They also tested the Acquity UPLC^®^ BEH C18 column, which provided satisfactory results for all target cytostatics, except 5FU. Therefore, the authors decided to develop a separate method for 5FU, considering the specificities of the molecule, using a Hypercarb^TM^ porous graphitic carbon column in combination with a mobile phase composed of water and MeOH, and a negative ionization detection mode [24]. Other authors also proposed the use of two analytical columns, one for hydrophobic cytostatics (e.g., PAC) and the other for hydrophilic ones (e.g., 5FU, DAC, and CYT), for the analysis of 20 cytostatics of different physico-chemical properties [16]. The Atlantis T3 is a silica-based, reversed-phase C18 column, and it was used to provide balanced retention of low-polar and hydrophobic compounds, whereas SeQuant ZIC-HILIC, a zwitterionic stationary phase, was determinant for the separation of polar and hydrophilic cytostatics [16].

### 6.2. Validation Parameters

The unequivocal identification and quantification of cytostatics in surfaces is a big challenge since the expected contamination levels are getting lower and lower, due to the introduction of protective and safety measures, but even those very low concentrations might result in health hazards, and should be detected. Since the exposure to cytotoxic compounds should be as low as possible, the IDLs of analytical methods should be the lowest possible as well. Different studies have suggested technical guidance values based on 50th, 75th, and 90th percentiles of positive contaminated samples. Kiffmeyer et al. proposed a substance-independent guidance value of 100 pg/cm^2^, based on the 90th percentile of the contamination values of eight cytostatics (CYC, DOC, ETO, 5FU, GEM, IFO, MET, PAC) [39]. The suitability of the analytical methodologies, available to monitor occupational exposure to cytostatics through surface sampling, will be discussed in regard to this guidance value.

Regarding the surface contamination methods, all but one study compiled in Table 3 proposed LC–MS/MS for the quantification of cytostatics. LC–MS/MS methods seem to exhibit similar performances, but it is difficult to compare them, as no consensual units of measurement for IDLs have been defined. Moreover, the results were not clearly presented and doubts exist if the IDL are the limits of detection for the instrumental method, assuming 100% recovery from the surface to convert into mass per square cm units, or if IDLs are the limits of detection of the overall analytical methodology and incorporate the different recoveries of cytostatics (also defined as method detection limit—MDL). In the cases where MDLs are provided, doubts about the recovery considered for its determination (recovery from surface, recovery from sampling device or matrix-matched extract recovery tests) further exist. Nonetheless, it seems that most IDLs are actually for the instrumental method; one reason for that is because a single IDL is reported for each cytostatic, independent on the sample collection/extraction procedure [11,19,20,22,24]. We recommend that both the IDL and the MDL should be reported, with all considerations applied (e.g., recovery values, solvent volume, sampled area explicitly indicated).

In Table 3, for the surface contamination methods, the units described by authors were harmonized by converting mass of cytostatic or mass per volume into mass of cytostatic per area. This unit of measurement further has the advantage of being more easily understood by the general public. The study of da Silva and co-workers did not provide the sampled area; therefore, an area of 100 cm^2^ (the most common among studies) was used to convert the units [11]. For the studies under review, the IDLs for CYC vary between 0.1 and 100 pg/cm^2^. The highest limit was reported by Müller-Ramírez et al., who proposed the determination of CYC, IFO, and PAC in surface extracts by LC in combination with ultraviolet detector [10]. The method comprises a SPE step before instrumental analysis, which allowed achieving tenfold concentration factors, but resulting IDLs were still slightly higher than those provided by LC–MS/MS. Despite the limitations of ultraviolet detector in terms of sensitivity and specificity, this instrumental method could be an excellent alternative to MS techniques, particularly for the assessment of occupational risks associated to cytostatics exposure in low-income countries or in healthcare settings/public laboratories, where advanced instrumental technologies may be unavailable or unaffordable. The IDLs of IFO (isomer of CYC) also ranged between 0.2 and 20 pg/cm^2^, with the highest value achieved by Müller-Ramírez et al. [10]. Similar IDL ranges were also reported for the epimers DOX and EPI (0.1–50 pg/cm^2^; Table 3). Anthracycline drugs, as DOX and EPI, are light sensitive and have the tendency to adsorb on glass surfaces. Thus, the authors recommend the use of light-blocking plastic materials to avoid any loss and consequently the increase of IDLs [20]. The highest IDLs reported in the target studies were for 5FU; values as high as 176 and 832 pg/cm^2^ [17,23]. Since these values are higher than the guidance value (100 pg/cm^2^), it seems plausible to state that the respective analytical methodologies are not compatible with the research of cytostatics traces in occupational environments. It should however be highlighted that 5FU could be detected on surfaces at concentrations above 0.3 pg/cm^2^, if the methods proposed by Dugheri et al. [16] or Acramel et al. [24] are followed. These authors developed a different separation and detection method for 5FU, due to its high polarity and solubility as well as its preferred deprotonation (negative ionization mode), compared to other target cytostatics. This targeted approach might have contributed for decreasing IDLs. Globally, the IDLs for all cytostatics ranged from 0.01 to 832 pg/cm^2^, with only two values above 100 pg/cm^2^ (those mentioned for 5FU).

Regarding air contamination, instrumental methods must be highly sensitive since airborne levels are expected to be very low. Indeed, Wakui et al. noticed the unsuitability of LC–UV for this purpose: quantification was not possible as the values obtained for real samples were close to the IDL for CYC [27]. It is worth noting that they reported a IDL 2000 times lower when employing LC–MS/MS (0.000005 µg/mL vs. 0.01 µg/mL for LC–UV; Table 3), using the same mobile phase, but a different stationary phase. Additionally, IDLs would ideally be expressed in mass of cytostatics per cubic meters of air sampled. The IDL from the GC–ECD method, reported by Panahi et al., fulfills those criteria [26]. It is however significantly higher than those previously reported in the literature for CYC (mostly HPLC–UV) [7]. On the other hand, the study by Wakui et al. defines the IDLs in terms of mass per volume of solvent. This poses a problem when method comparison is intended, similarly to what was discussed above for surface contamination methods.

Other validation parameters were also reported and discussed in some of the studies: linearity, selectivity, precision, and accuracy. The selectivity is one of the most critical elements of a method validation procedure, but few studies have reported their evaluation. The selectivity is defined as the ability of the method to differentiate and quantify the target analytes, even in the presence of interfering compounds, either coming from the matrix or other sources. It has been evaluated by performing blank tests (wipe samples without cytostatics) and checking for the presence of any extraneous peaks near the retention times of the target cytostatics. To date, no interference was detected or the signals of the interfering peaks coeluting with the target analytes were found to be insignificant [11,21,22,24]. Intra- and inter-day precisions (expressed as coefficient of variation in percentage) were found to be lower than 15% in all studies, except for MIT in the study of Dal Bello and co-workers [21]. The research group verified that MIT underwent a hydrolysis of the aziridinic ring and, as a consequence of its degradation, the precision, accuracy, and recovery parameters were affected, resulting in values out of the acceptable ranges [21]. With regard to accuracy, it must be noted that no certified materials are available for cytostatics. Thus, the standard addition method should be used for evaluating the reliability of the methods. Generally, a deviation of ±15% from the expected concentration is set, except in the vicinity of the limits of quantification and detection, where a deviation of ±20% is considered acceptable. As can be found in Table 3, some studies did not measure the accuracy, but the available values are within the acceptable range.

It is important to emphasize that none study to date has determined the global uncertainty associated to the results, which is a crucial information for the correct interpretation of results from method application in healthcare settings. It is particularly relevant when comparing results from different methods or when a proposed maximum limit is under consideration.

## 7. Conclusions

Following previous recommendations, recent methods for monitoring surface contamination of cytostatics in healthcare settings have been improved. Indeed, cost and time-saving sampling, sample preparation, and instrumental procedures have been used. Most studies have also developed multi-compound methods in order to avoid underestimation of the potential exposure risk of people connected to the cytostatic circuit. Moreover, very low IDLs have been attained for many of the cytostatics studied (in the level of pg/cm^2^), in the wake of proposals to use the ALARA principle to reduce environmental contamination (as no level of exposure has been considered safe). For the studies under review, the IDLs ranged from 0.01 to 832 pg/cm^2^, with only two values above 100 pg/cm^2^ (for 5FU). Regarding methods for air contamination assessment, no substantial advances have been observed. Although inhalation of aerosolized cytostatics might happen in some (rather specific) situations, this exposure route is not normally critical, and has been the focus of very few studies.

However, a lot could still be further improved in the procedures for monitoring of both surface and air contamination. Although some elements of sampling and sample preparation have not been extensively explored, other considerations regarding the whole process should have a bigger focus on future studies. Sample preservation and stability during shipping and storage (both before and after sample preparation), cytostatics’ stability in surfaces (possibility of accumulation), and recovery studies from several specific surfaces are some relevant examples. Furthermore, we emphasize the importance of past recommendations to include complete validation assessments in studies of this topic, since they are still scarcely described. Specifically, reporting precision, accuracy, and both IDLs and MDLs, unambiguously. In order to achieve a standardized and reliable overall procedure, all these and some other elements need to be considered. We further recommend the use of standardized reporting units (ng/cm^2^ for wipe sampling and ng/m^3^ for air sampling) and the estimation of global uncertainty associated to the results to avoid misleading interpretations of results.

## Figures and Tables

**Table 1:** Cytostatics studied by the reviewed analytical methods for environmental contamination and their chemical structures [31,32].

Cytostatic	Abbreviation	Chemical Structure	Studied by
5-Fluorouracil	5FU	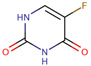	[11,12,13,14,15,16,17,19,21,23,24,25]
Busulfan	BUS	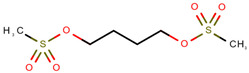	[12,13,14]
Carboplatin	CPt	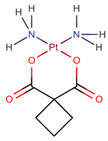	[21]
Cyclophosphamide	CYC	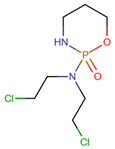	[10,11,12,13,14,15,16,17,18,19,20,21,23,24,25,26,27]
Cytarabine	CYT	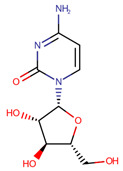	[12,13,14,15,16,21,24]
Dacarbazine	DAC	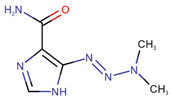	[12,13,14,15,16,24]
Daunorubicin	DAU	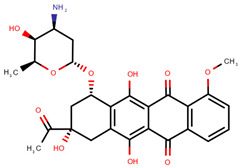	[12,13,14]
Docetaxel	DOC	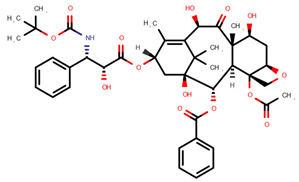	[11,12,13,14,15,16,17,18,20,24]
Doxorubicin	DOX	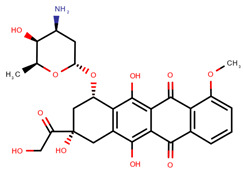	[11,12,13,14,15,16,18,19,20,21,24,25]
Epirubicin	EPI	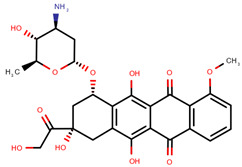	[12,13,14,15,16,18,20,24,25]
Etoposide	ETO	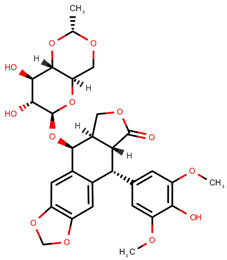	[12,13,14,15,16,18,24]
Etoposide phosphate	ETP	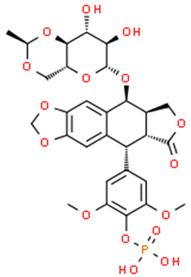	[12,13,14]
Fludarabine phosphate	FLU	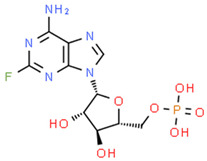	[12,13,14]
Fotemustine	FOT	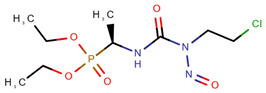	[15,16]
Ganciclovir	GAN	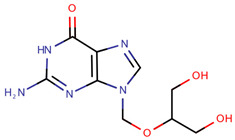	[12,14]
Gemcitabine	GEM	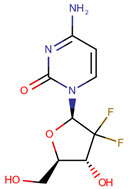	[12,13,14,15,16,18,21,24,25]
Idarubicin	IDA	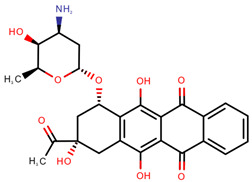	[12,13,14,15,16]
Ifosfamide	IFO	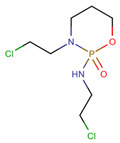	[10,12,13,14,15,16,17,18,20,21,24,25]
Irinotecan	IRI	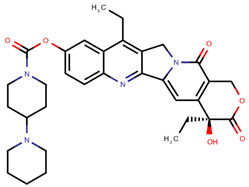	[12,13,14,15,16,18,20,24]
Melphalan	MEL	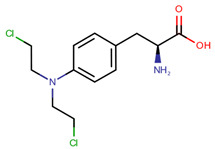	[15,16]
Methotrexate	MET	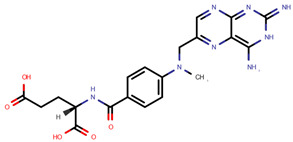	[12,13,14,15,16,17,18,21,23,24]
Mitomycin C	MIT	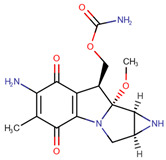	[15,16,21,22]
Oxaliplatin	OPt	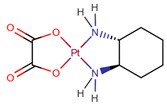	[17,23]
Paclitaxel	PAC	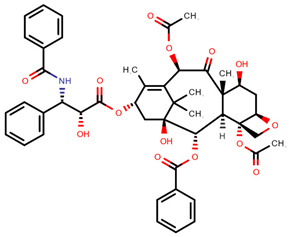	[10,12,13,14,15,16,17,18,20,23,24]
Pemetrexed	PEM	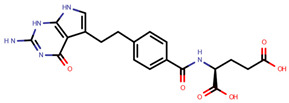	[12,13,14,24]
Raltitrexed	RAL	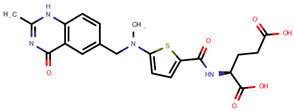	[12,13,14]
Topotecan	TOP	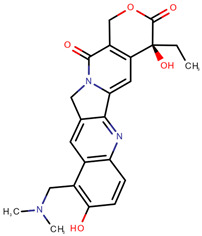	[12,13,14,15,16,18]
Vinblastine	VBL	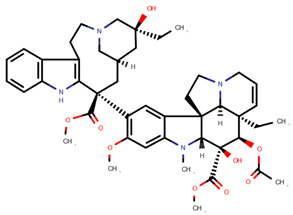	[12,13,14,15,16,17]
Vincristine	VCR	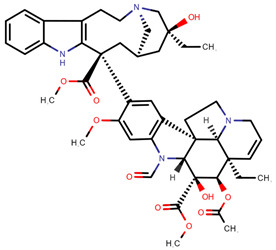	[12,13,14,15,16,17,20,23]
Vindesine	VDE	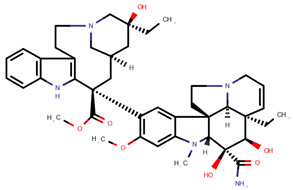	[17,20]
Vinorelbine	VOR	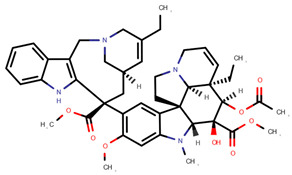	[12,13]

**Table 2:** Sampling and sample preparation parameters for environmental monitoring of surface contamination with cytostatics, and recoveries.

Sampling					Sample Preparation			Recovery (%)		Cytostatic	References
Surface Type	Sampling Area (cm^2^)	Sampling Device	Wetting Solution	Wiping Procedure	Extraction Solution	Extraction Procedure	Removal of Suspended Solids	From Surfaces	From Sampling Devices		
N/S	400	Cellulose filter paper (Whatman 42, 55 mm)	MeOH:ACN:potassium phosphate buffer (25:10:65; 0.25 mL)	2 wetted wipes	9.75 mL MeOH:ACN: potassium phosphate buffer (25:10:65)	Sonication, 10 min	Filtration (0.22 µm); SPE; evaporate under nitrogen; reconstitute	N/S	94 96 89	CYC IFO PAC	[10]
Formica^®^ Gloves Door locks	N/S	Wipe (Kleenex tissues)	MeOH:ethyl acetate (1:2; 1 mL)	1 wetted wipe; vertical and horizontal wiping	4 mL MeOH:ethyl acetate (1:2)	Ultrasonication, 30 min	N/S	105 103 102 61	N/S	5FU CYC DOC DOX	[11]
Stainless steel	100	Polyester swab (Texwipe^®^ TX716)	ISO:H_2_O (75:25)	1 wetted swab	10 mM acetic acid (pH 5.1, with 2% ACN)	Vortex, 5 min	N/S	N/S	95 (mean value)	5FU BUS CYC CYT DAC DAU DOC DOX EPI ETO ETP FLU GAN GEM IDA IFO IRI MET PAC PEM RAL TOP VCR	[12,13,14]
Stainless steel	100	Polyester swab (Texwipe^®^ TX716)	ISO:H_2_O 75:25)	1 wetted swab	10 mM acetic acid (pH 5.1, with 2% ACN)	Sonication, 5 min	N/S	N/S	95 (mean value)		
Stainless steel	100	Polyester swab (Texwipe^®^ TX714)	ISO:H_2_O (75:25)	1 wetted swab	10 mM acetic acid (pH 5.1, with 2% ACN)	Vortex, 5 min	N/S	N/S	90 (mean value)		
Stainless steel	100	Polyester swab (Texwipe^®^ TX714)	ISO:H_2_O (75:25)	1 wetted swab	10 mM acetic acid (pH 5.1, with 2% ACN)	Sonication, 5 min	N/S	N/S	90 (mean value)		
Stainless steel	100	Polyurethane swab (Texwipe^®^ TX712)	ISO:H_2_O (75:25)	1 wetted swab	10 mM acetic acid (pH 5.1, with 2% ACN)	Vortex, 5 min	N/S	N/S	84 (mean value)		
Stainless steel	100	Polyurethane swab (Texwipe^®^ TX712)	ISO:H_2_O (75:25)	1 wetted swab	10 mM acetic acid (pH 5.1, with 2% ACN)	Sonication, 5 min	N/S	N/S	74 (mean value)		
Stainless steel	100	Cellulose filter paper (Whatman, 3mm)	ISO:H_2_O (75:25)	1 wetted filter	10 mM acetic acid (pH 5.1, with 2% ACN)	Vortex, 5 min	N/S	N/S	69 (mean value)		
Stainless steel	100	Cellulose filter paper (Whatman, 3mm)	ISO:H_2_O (75:25)	1 wetted filter	10 mM acetic acid (pH 5.1, with 2% ACN)	Sonication, 5 min	N/S	N/S	69 (mean value)		
Stainless steel	100	Cellulose filter paper (Whatman, 3mm)	ISO:H_2_O (75:25)	1 wetted filter	2 mL diluent	Vortex, few seconds	N/S	N/S	N/S		
Stainless steel Non-porous polycarbonate Polyvinyl chloride flooring	400	Wipe (3-layer nonwoven, STS Medical Group Luigi Salvadori)	H_2_O:MeOH (equimolar; 0.5 mL/filter)	1 wetted wipe; right, down and left wiping	1.8 mL H_2_O:MeOH (equimolar)	Automated wipe desorption (Chromline)	N/S	N/S	70–77 85–94 83–91 81–89 82–89 83–91 86–96 79–94 85–88 75–81 76–88 84–95 86–92 83–90 87–91 87–93 78–91 84–93 87–94 79–89 75–84	5FU CYC CYT DAC DOC DOX EPI ETO FOT GEM IDA IFO IRI MEL MET MIT PAC Pt TOP VBL VCR	[15,16]
Stainless steel Non-porous polycarbonate Polyvinyl chloride flooring	400	Wipe (3-layer nonwoven, STS Medical Group Luigi Salvadori)	H_2_O:MeOH (equimolar; 0.5 mL/filter)	1 wetted wipe; right, down and left wiping	2 mL H_2_O:MeOH (equimolar)	Filtered in-line (0.2 µm filters; manually or in automated mode)	N/S				
Stainless steel 304 (new)	100	Cellulose filter paper (Whatman 42, 70 mm)	H_2_O:MeOH (20:80, 0.1% HCOOH; 0.5 mL/filter)	1 wetted filter; vertical and horizontal wiping	5 mL H_2_O:MeOH (50:50, 0.1% HCOOH)	Ultrasonication, 35 min	Centrifugation (4500 rpm, 15 min)	~40	44	5FU	[17]
								~95	101	CYC	
								~100	113	DOC	
								~100	101	IFO	
								~70	100	MET	
								~60	73	OPt	
								~95	98	PAC	
								~80	98	VBL	
								~85	95	VCR	
								~70	97	VDE	
N/S	900	Wipe (Kimwipe^®^)	H_2_O:ISO (30:70; 1 mL/wipe)	3 wetted wipes; each in a different direction (right, down, left)	30 mL H_2_O:ISO (70:30, 0.1% HCOOH)	Ultrasonication, 15 min	Filtration (cellulose, 0.45 µm; 1 mL extract)	N/S	~110	CYC	[18]
									~110	DOC	
									~85	DOX	
									~90	EPI	
									~120	ETO	
									~105	GEM	
									~115	IFO	
									~105	IRI	
									~120	MET	
									~110	PAC	
									~100	TOP	
N/S	900	Wipe (Kimwipe^®^)	H_2_O:ISO (30:70; 1 mL/wipe)	3 wetted wipes; each in a different direction	30 mL H_2_O:ISO (80:20, 0.1% HCOOH)	Ultrasonication, 15 min	Filtration (cellulose, 0.45 µm; 1 mL extract)	N/S	55 (DOX, EPI) –100 (DOC, IFO)	CYC DOC DOX EPI ETO GEM IFO IRI MET PAC TOP	[18]
N/S	900	Wipe (Kimwipe^®^)	H_2_O:ISO (30:70; 1 mL/wipe)	3 wetted wipes; each in a different direction	30 mL H_2_O:ISO (85:15, 0.1% HCOOH)	Ultrasonication, 15 min	Filtration (cellulose, 0.45 µm; 1 mL extract)	N/S	40 (DOX, EPI) –95 (IFO)		
N/S	900	Wipe (Kimwipe^®^)	H_2_O:ISO (30:70; 1 mL/wipe)	3 wetted wipes; each in a different direction	30 mL H_2_O:ISO (90:10, 0.1% HCOOH)	Ultrasonication, 15 min	Filtration (cellulose, 0.45 µm; 1 mL extract)	N/S	35 (DOX, EPI) –105 (IFO)		
N/S	900	Wipe (Kimwipe^®^)	H_2_O:ISO (30:70; 1 mL/wipe)	3 wetted wipes; each in a different direction	30 mL H_2_O:ISO (95:5, 0.1% HCOOH)	Ultrasonication, 15 min	Filtration (cellulose, 0.45 µm; 1 mL extract)	N/S	25 (DOX, EPI)– 100 (DOC, ETO)		
N/S	900	Wipe (Kimwipe^®^)	H_2_O:ISO (30:70; 1 mL/wipe)	3 wetted wipes; each in a different direction	30 mL H_2_O:ACN (80:20, 0.1% HCOOH)	Ultrasonication, 15 min	Filtration (cellulose, 0.45 µm; 1 mL extract)	N/S	70 (EPI) –100 (PAC)		
N/S	900	Wipe (Kimwipe^®^)	H_2_O:ISO (30:70; 1 mL/wipe)	3 wetted wipes; each in a different direction	30 mL H_2_O:ACN (85:15, 0.1% HCOOH)	Ultrasonication, 15 min	Filtration (cellulose, 0.45 µm; 1 mL extract)	N/S	55 (EPI)– 90 (IFO, CYC)		
N/S	900	Wipe (Kimwipe^®^)	H_2_O:ISO (30:70; 1 mL/wipe)	3 wetted wipes; each in a different direction	30 mL H_2_O:ACN (90:10, 0.1% HCOOH)	Ultrasonication, 15 min	Filtration (cellulose, 0.45 µm; 1 mL extract)	N/S	40 (EPI) –105 (ETO)		
N/S	900	Wipe (Kimwipe^®^)	H_2_O:ISO (30:70; 1 mL/wipe)	3 wetted wipes; each in a different direction	30 mL H_2_O:ACN (95:5, 0.1% HCOOH)	Ultrasonication, 15 min	Filtration (cellulose, 0.45 µm; 1 mL extract)	N/S	30 (EPI) –105 (ETO)		
N/S	900	Wipe (Kimwipe^®^)	H_2_O:ISO (30:70; 1 mL/wipe)	3 wetted wipes; each in a different direction	30 mL H_2_O:DMSO (80:20, 0.1% HCOOH)	Ultrasonication, 15 min	Filtration (cellulose, 0.45 µm; 1 mL extract)	N/S	45 (EPI) –105 (DOC)		
N/S	900	Wipe (Kimwipe^®^)	H_2_O:ISO (30:70; 1 mL/wipe)	3 wetted wipes; each in a different direction	30 mL H_2_O:DMSO (85:15, 0.1% HCOOH)	Ultrasonication, 15 min	Filtration (cellulose, 0.45 µm; 1 mL extract)	N/S	35 (EPI) –115 (ETO)		
N/S	900	Wipe (Kimwipe^®^)	H_2_O:ISO (30:70; 1 mL/wipe)	3 wetted wipes; each in a different direction	30 mL H_2_O:DMSO (90:10, 0.1% HCOOH)	Ultrasonication, 15 min	Filtration (cellulose, 0.45 µm; 1 mL extract)	N/S	25 (EPI)– 95 (CYC, IFO)		
N/S	900	Wipe (Kimwipe^®^)	H_2_O:ISO (30:70; 1 mL/wipe)	3 wetted wipes; each in a different direction	30 mL H_2_O:DMSO (95:5, 0.1% HCOOH)	Ultrasonication, 15 min	Filtration (cellulose, 0.45 µm; 1 mL extract)	N/S	25 (EPI) –100 (CYC)		
Stainless steel	100	Cellulose filter paper (Whatman 42, 55 mm)	Sterile water (10 mL)	1 wetted filter, then 1 dry filter	15 mL acetic acid 1%	Mix 10 min; SPE	Centrifugation (10,000 rpm, 4 °C; 50 mL acetic acid 1%)	N/S	76.3 76.3 70.0	5FU CYC DOX	[19]
N/S	800	Wipe (Kimwipe^®^ S-200)	MeOH (70%, 0.1% HCOOH; 1 mL/wipe)	3 wetted wipes	8 mL MeOH (70%, 0.1% HCOOH)	Shaker, 30 min, 2000 rpm	N/S	N/S	N/S	CYC, DOC, DOX, EPI, IFO, IRI, PAC, VBL, VCR, VDE	[20]
N/S	2000	Swab (sterile, non-woven)	MeOH:(0.05% HCOOH) (8:2; 4 mL)	1 wetted swab (wipe twice)	40 mL H_2_O (0.05% HCOOH)	Ultrasonication, 15 min	N/S	N/S	97	5FU	[21]
									99	CPt	
									101	CYC	
									93	CYT	
									82	DOX	
									82	GEM	
									92	IFO	
									87	MET	
									64	MIT	
Stainless steel 304	100	Cellulose filter paper (Whatman 42, 55 mm)	ACN:ISO:H_2_O (20:45:35; 0.2 mL/filter)	3 dry filters (sampling area wetted each time)	9 mL ACN:ISO:H_2_O (20:45:35)	Orbital shaker, 30 min	Filtration (PVDF, 0.22 µm)	62–98	N/S	MIT	[22]
Stainless steel 304	100	Polyester swab (Texwipe^®^ TX714A)	ACN:ISO:H_2_O (20:45:35; 0.2 mL/filter)	3 dry filters (sampling area wetted each time)	9 mL ACN:ISO:H_2_O (20:45:35)	Orbital shaker, 30 min	Filtration (PVDF, 0.22 µm)	61–98	N/S		
Vinyl	100	Cellulose filter paper (Whatman 42, 55 mm)	ACN:ISO:H_2_O (20:45:35; 0.2 mL/filter)	3 dry filters (sampling area wetted each time)	9 mL ACN:ISO:H_2_O (20:45:35)	Orbital shaker, 30 min	Filtration (PVDF, 0.22 µm)	51–63	N/S		
Vinyl	100	Polyester swab (Texwipe^®^ TX714A)	ACN:ISO:H_2_O (20:45:35; 0.2 mL/filter)	3 dry filters (sampling area wetted each time)	9 mL ACN:ISO:H_2_O (20:45:35)	Orbital shaker, 30 min	Filtration (PVDF, 0.22 µm)	53–62	N/S		
Formica^®^	100	Cellulose filter paper (What-man 42, 55 mm)	ACN:ISO:H_2_O (20:45:35; 0.2 mL/filter)	3 dry filters (sampling area wetted each time)	9 mL ACN:ISO:H_2_O (20:45:35)	Orbital shaker, 30 min	Filtration (PVDF, 0.22 µm)	30–96	N/S		
Formica^®^	100	Polyester swab (Texwipe^®^ TX714A)	ACN:ISO:H_2_O (20:45:35; 0.2 mL/filter)	3 dry filters (sampling area wetted each time)	9 mL ACN:ISO:H_2_O (20:45:35)	Orbital shaker, 30 min	Filtration (PVDF, 0.22 µm)	63–97	N/S		
Stainless steel (new and worn)	100	Cellulose filter paper (Whatman 42, 70 mm)	H_2_O:MeOH (20:80, 0.1% HCOOH; 0.5 mL)	1 wetted filter; vertical and horizontal wiping	5 mL H_2_O:MeOH (50:50, 0.1% HCOOH)	Ultrasonication, 35 min	Centrifugation (4500 rpm, 15 min); filtration	~60 ~80 ~20 ~40 ~85 ~60	63 99 102 80 101 103	5FU CYC MET OPt PAC VCR	[23]
Stainless steel (new and worn)	100	Cellulose filter paper (Whatman 42, 70 mm)	H_2_O:MeOH (20:80, 0.1% HCOOH; 0.5 mL)	1 wetted filter; vertical and horizontal wiping	5 mL H_2_O:MeOH (20:80, 0.1% HCOOH)	Ultrasonication, 35 min	Centrifugation (4500 rpm, 15 min); filtration	-	65 (5FU) – 120 (PAC)	5FU CYC MET OPt PAC VCR	
Stainless steel (new and worn)	100	Cellulose filter paper (Whatman 42, 70 mm)	H_2_O:MeOH (20:80, 0.1% HCOOH; 0.5 mL)	1 wetted filter; vertical and horizontal wiping	5 mL H_2_O:MeOH (50:50, 0.1% HCOOH)	Ultrasonication, 20 min	Centrifugation (4500 rpm, 15 min); filtration	-	63 (5FU) – 99 (VCR)		
Stainless steel (new and worn)	100	Cellulose filter paper (Whatman 42, 70 mm)	H_2_O:MeOH (80:20, 0.1% HCOOH; 0.5 mL)	1 wetted filter; vertical and horizontal wiping	5 mL H_2_O:MeOH (50:50, 0.1% HCOOH)	Ultrasonication, 35 min	Centrifugation (4500 rpm, 15 min); filtration	25 (VCR) –95 (CYC)	N/S		
Stainless steel (new and worn)	100	Cellulose filter paper (Whatman 42, 70 mm)	H_2_O:MeOH (50:50, 0.1% HCOOH; 0.5 mL)	1 wetted filter; vertical and horizontal wiping	5 mL H_2_O:MeOH (50:50, 0.1% HCOOH)	Ultrasonication, 35 min	Centrifugation (4500 rpm, 15 min); filtration	20 (MET) –70 (CYC)	N/S		
Stainless steel (new and worn)	100	Cellulose filter paper (Whatman 42, 70 mm)	H_2_O (0.1% HCOOH; 0.5 mL)	1 wetted filter; vertical and horizontal wiping	5 mL H_2_O:MeOH (50:50, 0.1% HCOOH)	Ultrasonication, 35 min	Centrifugation (4500 rpm, 15 min); filtration	10 (PAC, VCR)– 70 (CYC)	N/S		
Stainless steel (new and worn)	100	Cellulose filter paper (Whatman 42, 70 mm)	MeOH (0.1% HCOOH; 0.5 mL)	1 wetted filter; vertical and horizontal wiping	5 mL H_2_O:MeOH (50:50, 0.1% HCOOH)	Ultrasonication, 35 min	Centrifugation (4500 rpm, 15 min); filtration	5 (MET)–80 (CYC, PAC)	N/S		
Stainless steel (new and worn)	100	Cellulose filter paper (Whatman 42, 70 mm)	H_2_O:MeOH:ACN (65:25:10, 0.1% HCOOH; 0.5 mL)	1 wetted filter; vertical and horizontal wiping	5 mL H_2_O:MeOH (50:50, 0.1% HCOOH)	Ultrasonication, 35 min	Centrifugation (4500 rpm, 15 min); filtration	30 (VCR)–95 (CYC)	N/S		
N/S	225	Viscose swab (DeltalaB)	Sterile water (0.05 mL)	1 wetted swab, then 1 dry swab; both in 3 different directions (vertical, horizontal, diagonal)	2 mL MeOH	Vortex, 30 s; ultrasonication, 10 min	Centrifugation (19,000 g, 5 min)	N/S	75–114	5FU, CYC, CYT, DAC, DOC, DOX, EPI, ETO, GEM, IFO, IRI, MET, PAC, PEM	[24]
N/S	100	Cellulose filter paper (Whatman, 55mm)	Sterile water (0.4 mL)	1 wetted filter, then 1 dry filter	15 mL acetic acid 1%	Mix 20 min; SPE	N/S	N/S	21	5FU	[25]
									75	CYC	
									18	DOX	
									14	EPI	
									54	GEM	
									81	IFO	

N/S—not specified; 5FU—5-fluorouracil; BUS—busulfan; CPt—carboplatin; CYC—cyclophosphamide; CYT—cytarabine; DAC—dacarbazine; DAU—daunorubicin; DOC—docetaxel; DOX—doxorubicin; EPI—epirubicin; ETO—etoposide; ETP—etoposide phosphate; FLU—fludarabine phosphate; FOT—fotemustine; GAN—ganciclovir; GEM—gemcitabine; IDA—idarubicin; IFO—ifosfamide; IRI—irinotecan; MEL—melphalan; MET—methotrexate; MIT—mitomycin C; OPt—oxaliplatin; PAC—paclitaxel; PEM—pemetrexed; Pt—platinum (as marker of cis-, carbo-, oxaliplatin); RAL—raltitrexed; TOP—topotecan; VBL—vinblastine; VCR—vincristine; VDE—vindesine; VOR—vinorelbine; H_2_O—water; MeOH—methanol; ACN—acetonitrile; ISO—isopropanol; HCOOH—formic acid; DMSO—dimethyl sulfoxide; SPE—solid-phase extraction; PVDF—polyvinylidene fluoride.

**Table 3:** Instrumental techniques used for environmental monitoring of surface and air contamination with cytostatics, and validation parameters.

Cytostatic	Instrumentation	Stationary Phase	Mobile Phase	Run Time	Linearity	Precision (%)	Accuracy (%) ^1^	IDL	References
CYC, IFO, PAC	LC–UV	Symmetry^®^ C18 (150 × 4.6 mm, 5 µm)	(A) H_2_O + 10 mM phosphate buffer (pH 6) (B) ACN	25 min	0.3–38 ng/cm^2^—PAC 0.6–38 ng/cm^2^—CYC 0.8–38 ng/cm^2^—IFO	Within-run: 1.5–1.7 Between-run: 1.7–2.3	N/S	20 pg/cm^2^—IFO 30 pg/cm^2^—PAC 100 pg/cm^2^—CYC	[10]
5FU, CYC, DOC, DOX	UPLC–MS/MS	SHIM-PACK XR-ODC-C18 (100 × 3 mm, 2.2 µm)	(A) H_2_O + 0.1% HCOOH (B) ACN	15 min	0.1–15 ng/cm^2^—5FU ^2^ 0.2–15 ng/cm^2^—CYC, DOC, DOX ^2^	Within-run: 1.2–14.2 Between-run: 1.3–13.2	N/S	25 pg/cm^2^—5FU ^2^ 50 pg/cm^2^—CYC, DOC, DOX ^2^	[11]
5FU, BUS, CYC, CYT, DAC, DAU, DOC, DOX, EPI, ETO, ETP, FLU, GAN, GEM, IDA, IFO, IRI, MET, PAC, PEM, RAL, TOP, VCR	UPLC–MS/MS	CORTECS UPLC T3 (100 × 2.1 mm, 1.6 µm)	(A) H_2_O + 10 mM ammonium acetate (pH 5.1) (B) ACN	17.5 min	N/S	N/S	N/S	N/S	[12,13]
5FU, CYC, CYT, DAC, DOC, DOX, EPI, ETO, FOT, GEM, IDA, IFO, IRI, MEL, MET, MIT, PAC, Pt, TOP, VBL, VCR	LC–MS/MS (all except Pt)	For 5FU, CYT, DAC, GEM: SeQuant ZIC-HILIC (100 × 2.1 mm, 5 µm)	For 5FU, CYT, DAC, GEM: (A) H_2_O + 0.1 M ammonium formate (B) ACN	15 min	0.003–0.1 ng/cm^2^ (all)	Within-run: 1.4–5.4 Between-run: 3.3–6.7	N/S	0.05 pg/cm^2^—Pt 0.2 pg/cm^2^—IFO 0.3 pg/cm^2^—CYC, EPI, MEL, PAC 0.4 pg/cm^2^—5FU, DOC, IRI, MET, TOP, VCR 0.5 pg/cm^2^—FOT 1.1 pg/cm^2^—IDA 1.2 pg/cm^2^—MIT 2.2 pg/cm^2^—GEM 2.3 pg/cm^2^—DOX, VBL 2.6 pg/cm^2^—CYT, DAC 3.0 pg/cm^2^—ETO	[15,16]
		For others: Atlantis T3 (100 × 2.1 mm, 3 µm)	For others: (A) H_2_O + 0.1% HCOOH (B) ACN:MeOH (60:40) + 0.1% HCOOH	20.5 min					
		For all (except Pt): YMC-Pack ODS-AQ (250 × 2.1 mm, 5 µm)	For all (except Pt): (A) H_2_O (B) ACN:MeOH (60:40) + 0.1% HCOOH	23 min					
	ICP–MS (Pt)	-	-	-					
5FU, CYC, DOC, IFO, MET, OPt, PAC, VBL, VCR, VDE	LC–MS/MS	Kinetex Biphenyl (50 × 4.6 mm, 2.6 µm)	(A) H_2_O (+formic acid + ammonium formate; pH 2.3) (B) MeOH	8 min	0.06–11 ng/cm^2^—5FU, OXP 0.01–11 ng/cm^2^—others	Within-run: 1–13 Between-run: 1–8	92–105	0.01 pg/cm^2^—VCR 0.04 pg/cm^2^—VBL 0.05 pg/cm^2^—PAC 0.4 pg/cm^2^—DOC 0.8 pg/cm^2^—VDE 1.8 pg/cm^2^—CYC 3.9 pg/cm^2^—MET 10.7 pg/cm^2^—IFO 86.9 pg/cm^2^—OPt 176.4 pg/cm^2^—5FU	[17]
CYC, DOC, DOX, EPI, ETO, GEM, IFO, IRI, MET, PAC, TOP	Micro-LC–MS/MS	YMC Triart C18 (50 × 0.3 mm, 1.9 µm)	(A) H_2_O + 0.1% HCOOH (B) ACN + 0.1% HCOOH	2.25 min	0.0004–0.4 ng/cm^2^—IFO 0.0004–1.8 ng/cm^2^—PAC 0.0004–4 ng/cm^2^—DOX 0.0004–0.9 ng/cm^2^—others	Within-run: 2.7–13.5 Between-run: 3.3–16.2	N/S	1.9 pg/cm^2^—DOX 2.2 pg/cm^2^—TOP 2.5 pg/cm^2^—CYC, GEM 3.5 pg/cm^2^—EPI 3.9 pg/cm^2^—IFO 4.0 pg/cm^2^—ETO 4.5 pg/cm^2^—MET 4.6 pg/cm^2^—IRI 7.6 pg/cm^2^—PAC 17.9 pg/cm^2^—DOC	[18]
5FU, CYC, DOX	LC–MS	Pursuit XRs Ultra (100 × 2 mm, 2.8 µm)	(A) H_2_O + 0.1% acetic acid (B) water ACN	30 min	0.01–1 ng/cm^2^—CYC 0.1–5—5FU, DOX	Within-run: 2.5–8.4 Between-run: 2.1–9.4	82–104	5 pg/cm^2^—CYC 50 pg/cm^2^—5FU, DOX	[19]
CYC, DOC, DOX, EPI, IFO, IRI, PAC, VBL, VCR, VDE	LC–MS/MS	Inertsil^®^ ODS-3 (50 × 2.1 mm, 3 µm)	(A) H_2_O + 0.1% HCOOH (B) ACN + 0.1% HCOOH	22 min	0.06–13 ng/cm^2^—VBL, VCR, VDE 0.06–1.3 ng/cm^2^—others	Within-run: 1.0–11.5 Between-run: 3.6–14.4	Within-run: 89–113 Between-run: 85–112	19 pg/cm^2^—VBL, VCR, VDE ^3^ 1.9 pg/cm^2^—others ^3^	[20]
5FU, CPt, CYC, CYT, DOX, GEM, IFO, MET, MIT	LC–HRMS/MS	Varian Pursuit C18 (150 × 2 mm, 3 µm)	Positive ion mode: (A) H_2_O + 0.05% HCOOH (B) MeOH Negative ion mode: (A) H_2_O + 0.1 mM ammonium acetate (B) MeOH	25 min	0.02–2 ng/cm^2^ (all)	Within-run: 5.0–12.2 (except MIT—99.9) Between-run: 3.0–7.5 (except MIT—19.9)	N/S	7 pg/cm^2^ (all) ^3^	[21]
MIT	LC–MS/MS	Zorbax Rx C18 (250 × 3.0 mm, 3.5 µm)	(A) ACN:H_2_O (10:90) + 0.1% acetic acid (B) ACN:H_2_O (75:25) + 0.1% acetic acid	24 min	0.2–50 ng/cm^2^	Stainless steel 304: 1.0–10.8 Vinyl: 2.3–12.6 Formica^®^: 0.7–8.5	Stainless steel 304: 93–104 Vinyl: 95–105 Formica^®^: 94–103	20 pg/cm^2^	[22]
5FU, CYC, MET, OPt, PAC, VCR	LC–MS/MS	Kinetex Biphenyl (50 × 4.6 mm, 2.6 µm)	(A) H_2_O (+ formic acid + ammonium formate; pH 2.3) (B) MeOH	7 min	0.01–11 ng/cm^2^—CYC, MET, PAC, VCR 0.6–11 ng/cm^2^—5FU, OPt	Within-run: 0.9–8.9 Between-run: 3.8–11.0	89–106	1 pg/cm^2^—PAC 4 pg/cm^2^—CYC, MET 5 pg/cm^2^—VCR 127 pg/cm^2^—OPt 832 pg/cm^2^—5FU	[23]
5FU, CYC, CYT, DAC, DOC, DOX, EPI, ETO, GEM, IFO, IRI, MET, PAC, PEM	LC–MS/MS	For 5FU: Hypercarb™ (100 × 2.1 mm, 5µm) For others: Acquity UPLC^®^ BEH C18 (50 × 2.1 mm, 1.9 µm)	For 5FU: (A) H_2_O (B) MeOH For others: (A) H_2_O (B) ACN + 0.1% HCOOH	8 min	0.0004–0.2 ng/cm^2^—CYC, DOX, EPI 0.0009–0.2 ng/cm^2^—5FU, DAC, GEM, IFO, IRI, MET, PAC, PEM 0.004–0.2 ng/cm^2^—CYT, DOC, ETO	1.5–13.5	61–133	0.1 pg/cm^2^—CYC, DOX, EPI ^3^ 1 pg/cm^2^—CYT, DOC, ETO ^3^ 0.3—others ^3^	[24]
5FU, CYC, DOX, EPI, GEM, IFO	UPLC–MS/MS	HSS T3 (50 × 2.1 mm, 1.8 µm)	(A) H_2_O + 0.5% acetic acid (B) ACN + 0.5% acetic acid	6.5 min	0.002–0.4 ng/cm^2^—CYC, GEM, IFO 0.025–2 ng/cm^2^—5FU, DOX, EPI	N/S	98–108	0.2 pg/cm^2^—CYC, GEM 0.4 pg/cm^2^—IFO 1.25 pg/cm^2^—5FU, DOX 5 pg/cm^2^—EPI	[25]
CYC	GC–ECD	BP5 (30 m)	Nitrogen (99.9995%)	25 min	212–1062 µg/m^3^	Within-run: 4.8 Between-run: 8.9	Within-run: 95–109 Between-run: 97–104	100 µg/m^3^	[26]
CYC	LC–UV	Inertsil^®^ ODS-3 (150 × 4.6 mm, 5 µm)	(A) H_2_O (B) ACN	N/S	0.05–500 µg/mL	N/S	N/S	0.01 µg/mL	[27]
CYC	LC–MS/MS	L-column 2 ODS (150 × 2.1 mm, 5 µm)	(A) H_2_O (B) ACN	N/S	0.00001–0.3 µg/mL	N/S	N/S	0.000005 µg/mL	

IDL—instrumental detection limit; N/S—not specified; 5FU—5-fluorouracil; BUS—busulfan; CPt—carboplatin; CYC—cyclophosphamide; CYT—cytarabine; DAC—dacarbazine; DAU—daunorubicin; DOC—docetaxel; DOX—doxorubicin; EPI—epirubicin; ETO—etoposide; ETP—etoposide phosphate; FLU—fludarabine phosphate; FOT—fotemustine; GAN—ganciclovir; GEM—gemcitabine; IDA—idarubicin; IFO—ifosfamide; IRI—irinotecan; MEL—melphalan; MET—methotrexate; MIT—mitomycin C; OPt—oxaliplatin; PAC—paclitaxel; PEM—pemetrexed; Pt—platinum (as marker of cis-, carbo-, oxaliplatin); RAL—raltitrexed; TOP—topotecan; VBL—vinblastine; VCR—vincristine; VDE—vindesine; VOR—vinorelbine; H_2_O—water; MeOH—methanol; ACN—acetonitrile; HCOOH—formic acid; LC–UV—liquid chromatography with ultraviolet detector; (UP)LC–MS/MS—(ultra-performance) liquid chromatography with tandem mass spectrometry; ICP–MS—inductively coupled plasma mass spectrometry; LC–MS—liquid chromatography with mass spectrometry; LC–HRMS/MS—liquid chromatography with tandem high resolution mass spectrometry; GC–ECD—gas chromatography with electron capture detector. ^1^ Recovery (%) in the matrix-matched extract. ^2^ Assuming 100 cm^2^ as sampling area. ^3^ Estimated from the limit of quantification, multiplying by 3/10.

## Data Availability

Data sharing not applicable.
